# A Comprehensive Prediction Model for Futile Recanalization in AIS Patients Post-Endovascular Therapy: Integrating Clinical, Imaging, and No-Reflow Biomarkers

**DOI:** 10.14336/AD.2024.0127

**Published:** 2024-04-25

**Authors:** Shuangfeng Huang, Jiali Xu, Haijuan Kang, Wenting Guo, Changhong Ren, Alexandra Wehbe, Haiqing Song, Qingfeng Ma, Wenbo Zhao, Yuchuan Ding, Xunming Ji, Sijie Li

**Affiliations:** ^1^Department of Neurology, Xuanwu Hospital, Capital Medical University, Beijing, China.; ^2^Department of Rehabilitation Medicine, Beijing Shijitan Hospital, Capital Medical University, Beijing, China.; ^3^Department of Neurology, Beijing Fengtai Hospital of Integrated Traditional Chinese and Modern Medicine, Beijing, China.; ^4^Beijing Key Laboratory of Hypoxic Conditioning Translational Medicine, Xuanwu Hospital, Capital Medical University, Beijing, China.; ^5^Department of Neurosurgery, Wayne State University School of Medicine, Detroit, MI, 48201, USA.; ^6^Beijing Institute of Brain Disorders, Laboratory of Brain Disorders, Ministry of Science and Technology, Collaborative Innovation Center for Brain Disorders, Capital Medical University, Beijing, China.; ^7^Department of Emergency, Xuanwu Hospital, Capital Medical University, Beijing, China.

**Keywords:** acute ischemic stroke, no-reflow phenomenon, D-dimer, contrast extravasation, neutrophil-to-lymphocyte ratio

## Abstract

Our study aimed to construct a predictive model for identifying instances of futile recanalization in patients with anterior circulation occlusion acute ischemic stroke (AIS) who achieved complete reperfusion following endovascular therapy. We included 173 AIS patients who attained complete reperfusion, as indicated by a Modified Thrombolysis in Cerebral Infarction (mTICI) scale score of 3. Our approach involved a thorough analysis of clinical factors, imaging biomarkers, and potential no-reflow biomarkers through both univariate and multivariate analyses to identify predictors of futile recanalization. The comprehensive model includes clinical factors such as age, presence of diabetes, admission NIHSS score, and the number of stent retriever passes; imaging biomarkers like poor collaterals; and potential no-reflow biomarkers, notably disrupted blood-brain barrier (OR 4.321, 95% CI 1.794-10.405; p = 0.001), neutrophil-to-lymphocyte ratio (NLR; OR 1.095, 95% CI 1.009-1.188; p = 0.030), and D-dimer (OR 1.134, 95% CI 1.017-1.266; p = 0.024). The model demonstrated high predictive accuracy, with a C-index of 0.901 (95% CI 0.855-0.947) and 0.911 (95% CI 0.863-0.954) in the original and bootstrapping validation samples, respectively. Notably, the comprehensive model showed significantly improved predictive performance over models that did not include no-reflow biomarkers, evidenced by an integrated discrimination improvement of 8.86% (95% CI 4.34%-13.39%; p < 0.001) and a categorized reclassification improvement of 18.38% (95% CI 3.53%-33.23%; p = 0.015). This model, which leverages the potential of no-reflow biomarkers, could be especially beneficial in healthcare settings with limited resources. It provides a valuable tool for predicting futile recanalization, thereby informing clinical decision-making. Future research could explore further refinements to this model and its application in diverse clinical settings.

## INTRODUCTION

Acute ischemic stroke (AIS) is a leading cause of morbidity and mortality worldwide. Endovascular thrombectomy (EVT) has emerged as an effective treatment for AIS patients with large vessel occlusion [1-5]. However, despite successful reperfusion, half of patients with complete reperfusion remain suffering poor long-term functional outcome, which is named as futile recanalization [6-8]. And the complete reperfusion after EVT is recognized as modified Thrombolysis in Cerebral Infarction (mTICI) scale of 3 (100% reperfusion). Predicting the risk of futile recanalization is instrumental in tailoring personalized patient management strategies and enhancing clinical outcomes. The development of a predictive model can facilitate the early identification of AIS patients at high risk of poor functional outcomes, even after achieving full reperfusion. This aids in making informed decisions regarding early intervention.

Numerous studies have attempted to identify clinical factors and imaging biomarkers to construct predictive models of futile recanalization. These models have primarily focused on AIS patients with successful reperfusion (mTICI of 2b-3), which signifies 50%-100% reperfusion in digital subtraction angiography (DSA) images [9-11]. However, recent studies have further revealed that AIS patients achieving an mTICI score of 3 have better prognosis compared to those with mTICI scores of 2b [12]. This finding underscores the limitations of previous models in accurately predicting futile recanalization in patients who achieve full reperfusion, thereby highlighting an urgent need for a more comprehensive and accurate prediction model. Given this context, our study aims to focus on AIS patients who not only achieve full reperfusion but also effective recanalization.

While several clinical and imaging factors associated with futile recanalization have been identified, including older age, diabetes, higher admission National Institute of Health Stroke Scale (NIHSS) score, more passes of stent retriever, and poor collateral status, models based solely on these factors have demonstrated limited predictive accuracy. In addition to the clinical factors, the mismatch between full reperfusion and unfavorable functional outcomes may be partially attributed to the incomplete microvascular reperfusion after complete recanalization of occluded large vessels, which is also known as “no-reflow phenomenon” and even approximately 30% patients with full reperfusion experienced [13]. Recent experimental studies have proved that cerebral no-reflow phenomenon exists during the first 24 hours after AIS [14]. Perfusion imaging methods are the main methods that are used to evaluate the reperfusion status after EVT, but these examinations are difficult to perform timely after EVT, which may render the prevention or counteraction of post-EVT no-reflow impossible.

Given these challenges, there is a need to identify alternative biomarkers that are more readily accessible and easier to measure within the first 24 hours post-AIS. Certain biomarkers such as inflammatory biomarkers, platelet profiles and permeability of blood-brain barrier (BBB) have been demonstrated to be no-reflow biomarkers in human heart or ischemic-reperfused animal brain [15-18]. Derived from the pathophysiologic pathways of microvascular no-reflow phenomenon, these biomarkers are postulated to predict futile recanalization in AIS [19]. These potential no-reflow biomarkers, which are more readily available and less resource-intensive to measure, could enhance the predictive accuracy of existing models and offer a practical solution for resource-limited settings.

Our study seeks to fill the existing gap by developing a comprehensive model that integrates clinical factors, imaging biomarkers, and potential no-reflow biomarkers for predicting futile recanalization in AIS patients who achieve full reperfusion. This model is expected to significantly contribute to personalized patient care and improve AIS management, particularly in healthcare institutions with limited resources.

## MATERIALS AND METHODS

### Study design and participants

This study was constructed at a high-volume stroke center of Xuanwu Hospital. Patients with AIS secondary to large vessels occlusion in the anterior circulation and treated with EVT from January 2019 to June 2021 were screened. All EVT procedures were performed following the Guidelines for the Early Management of Patients with Acute Ischemic Stroke [20, 21]. The inclusion criteria for this study were as follows: (1) aged≥18 years at the stroke onset; (2) AIS caused by middle cerebral artery (MCA) occlusion in the M1 segment or intracranial internal carotid artery (ICA) and treated with EVT; (3) the time from onset to puncture was limited in 24 hours; (3) the culprit vessel defined as mTICI score of 3 was completely recanalized as confirmed on the last DSA image.

Participants were excluded from our study based on the following criteria: (1) unknown 3-month Modified Rankin Scales (mRS) score; (2) mRS > 2 before current AIS; (3) re-occlusion of the corresponding artery after EVT within 72 hours; (4) absence of non-contrast computed tomography (NCCT) and computed tomography angiography (CTA) images on admission before EVT; (5) absence of dual-energy CT images within 24 hours after admission to define contrast extravasation; (6) absence of blood examination before EVT. This study was approved by the ethic committee of Xuanwu Hospital of Capital Medical University, and all patients or their legally authorized representatives provided written informed consent upon admission to hospital.

### Evaluation of clinical factors

Demographic and clinical characteristics were collected. Comorbidities including hypertension, diabetes, hyperlipidemia, smoking status, atrial fibrillation and previous stroke were also documented. Stroke severity was evaluated using the NHISS and Alberta Stroke Program Early Computed Tomography Score (ASPECTS). Stroke etiology was classified using TOAST (Trial of Org 10 172 in Acute Stroke Treatment) criteria. All enrolled patients were assessed for mRS at 3 months post-AIS by experienced neurologists. Futile recanalization and effective recanalization were defined as mRS of 3-6 and mRS of 0-2 at 3 months after achieving complete reperfusion, respectively [22].

### Evaluation of imaging biomarkers

Global cortical atrophy (GCA) reported to correlated with futile recanalization was quantitatively measured based on admission NCCT images to evaluate brain atrophy, and its assessments were performed according to previous studies [22]. Collateral circulation was assessed on the admission CTA images based on the following scoring: 0 (absent collaterals of the ischemic area); 1 (collateral filling<50% of the ischemic area); 2 (collateral filling 50%~99% of the ischemic area); and 3 (collateral filling 100% of the ischemic area). Poor collateral circulation was defined as scores ranging from 0-1[23]. All imaging was assessed by two stroke neurologists independently, and any disagreement was resolved by reaching a consensus between the two of them, if no consensus could be reached, a third stroke neurologist had the final decision.

### Evaluation of potential biomarkers of no-reflow phenomenon

Biomarkers including inflammatory markers, platelet profiles which have been reported to correlate with cardiovascular no-reflow phenomenon was selected as potential no-reflow markers [17]. In addition, BBB disruption (contrast extravasation) and components of the fibrinolysis-coagulation system (plasma D-dimer and fibrinogen) that were reported to play critical roles in microvascular obstruction in brains of baboons with cerebral ischemia/reperfusion were also selected as candidates [24]. And these biomarkers should be collected within 24 hours after admission.

Early contrast extravasation was evaluated to detect the integrity of the BBB. Dual-energy CT examination was performed within 24 hours after admission to assess contrast extravasation and hemorrhagic transformation. The detailed procedure of dual-energy CT scan was described at previous studies. To differentiate between intracranial hemorrhagic transformation and contrast extravasation, virtual images and iodine overlay images were compared. Contrast extravasation was determined by iodine contrast exhibiting on the iodine overlay image rather than the virtual non-contrast image, while hemorrhagic transformation was determined by blood being mapped on the virtual non-contrast image but not the iodine overlay image. Hemorrhagic transformation was then confirmed by comparing the NCCT scan at 72 hours after admission with the previous CT scan.

Blood samples were collected on admission prior to EVT procedures. An automated blood cell counter (MEK-7222K, NIHON KOHEN, JAPAN) was applied to determine the absolute leukocyte, neutrophil, lymphocyte, monocyte, platelet counts, mean platelet volume, and platelet distribution width. Plasma fibrinogen and D-dimer levels were measured by an automated coagulation analyzer (CK2651, GE company, USA) with their corresponding reagents. Cutoff values for these blood biomarkers were determined using reference intervals predetermined by the Laboratory of Xuanwu Hospital, Capital Medical University (Supplementary Table 1).

Inflammatory profiles including neutrophil-to-lymphocyte ratios (NLR) and platelet-to-lymphocyte ratios (PLR) were calculated by dividing the absolute count of neutrophils and platelets by the absolute count of lymphocytes, respectively [25]. Systemic inflammatory response index (SIRI) was calculated by the formula: (absolute neutrophil count × absolute monocyte count)/absolute lymphocyte count [26]. Platelet profiles including platelet counts, mean platelet volume, and platelet distribution width were also recorded. Coagulation factors including D-dimer and fibrinogen were also collected.

### Statistical analysis

The statistical analysis was conducted using SPSS Statistics Version 23 (IBM, Armonk, New York) and R statistical software (version 4.1.2). Univariate analyses were conducted to assess baseline characteristics between two groups (futile recanalization vs. effective recanalization). Continuous variables were analyzed using independent sample t-tests or Mann-Whitney U tests and were described as means ± SD or medians (interquartile range, IQR). Categorical variables were represented as n (%) and analyzed using chi-squared tests or Fisher exact tests.

A multivariate logistic regression model with stepwise forward method was used to select significant variables (p < 0.05 at univariate analysis). The odds ratio (OR) and 95% confidence interval (CI) of each significant variable was calculated and recorded. Model 1 was established by significant clinical factors and imaging biomarkers without no-reflow variables. Model 2 was a comprehensive model incorporating significant clinical factors, imaging biomarkers and no-reflow variables, which was exhibited as a nomogram. The concordance index (C-index) was calculated to assess the ability of discrimination of the comprehensive model using “rms” package. C-index>0.75 is considered to indicate reliable discrimination. Calibration plot was used to assess the calibration. In addition, the model was validated internally by bootstraps of 1000 resamples. Decision curve analysis was performed by “rmda” package to evaluate the clinical usefulness of the nomogram.

The additional predictive value of no-reflow markers was evaluated by area under curve (AUC), integrated discrimination improvement (IDI), and categorized reclassification improvement (NRI) analyses comparing the comprehensive model to the model without no-reflow biomarkers. IDI was used to determine the increase in discrimination of futile recanalization and effective recanalization. NRI was applied to quantify correct reclassification when adding no-reflow markers to the basic model. A p < 0.05 was considered significant for all analyses.

**Table 1:** Baseline characteristics of patients with mTICI 3.

Factors	Total number (n=173)	Effective recanalization (mRS 0-2) (n=97)	Futile recanalization (mRS 3-6) (n=76)	P value
**Demographics**				
**Age (y)**	65.27±12.83	61.32±13.10	70.26±10.61	<0.001^*^
**Male, n (%)**	106(61.3%)	69(71.1%)	37(46.6%)	0.003^*^
**Vascular risk factors**				
**Hypertension, n (%)**	117(67.6%)	64(66.0%)	53(69.7%)	0.600
**Diabetes, n (%)**	58(33.3%)	21(21.6%)	37(48.7%)	<0.001^*^
**Hyperlipidemia, n (%)**	84(48.6%)	49(50.5%)	35(46.1%)	0.560
**Smoking, n (%)**	51(29.7%)	34 (35.4%)	17(22.4%)	0.063
**Atrial fibrillation, n (%)**	72(41.6%)	36(37.1%)	36(47.4%)	0.174
**Previous stroke**	35(20.2%)	13(13.4%)	22(28.9%)	0.012^*^
**Admission characteristics**				
**Admission SBP (mmHg)**	145.79±22.15	143.03±20.23	149.32±24.06	0.064
**Admission DBP (mmHg)**	84.10±17.6	83.75±17.25	84.54±18.16	0.771
**NIHSS, median (IQR)**	15(12-18)	13(10-17)	17(14-20)	<0.001^*^
**ASPECTS, median (IQR)**	9(7-10)	9(8-9)	9(7-10)	0.811
**ICA occlusion, n (%)**	60(34.7%)	30(30.9%)	30(39.5%)	0.241
**MCA occlusion, n (%)**	113(65.3%)	67(69.1%)	46(60.5%)	
**FBG (mmol/l)**	8.02±2.99	7.33±2.79	8.91±3.01	0.001^*^
**Stroke etiology**				0.129
**LAA, n (%)**	76(43.9%)	47(48.5%)	29(38.2%)	
**CE, n (%)**	86(49.7%)	42(43.3%)	44(57.9%)	
**Others, n (%)**	11(6.4%)	8(8.2%)	3(3.9%)	
**Treatment**				
**Using stent retriever**	106(61.3%)	52(53.6%)	54(71.1%)	0.019^*^
**Passes of stent retriever, median (IQR)**	1(0-1)	1(0-1)	1(0-2)	0.006^*^
**OTP, mins, median (IQR)**	397.5(285.7-572.0)	425.0(325.3-600.0)	371.0(255.0-522.0)	0.224
**OTR, mins, median (IQR)**	438.0(320.0-596.8)	474.0(345.0-613.8)	406.0(308.0-562.5)	0.134
**General anesthesia, n (%)**	36(20.8%)	17(17.5%)	19(25%)	0.229
**Thrombolysis i.v., n (%)**	71(41.0%)	34(35.1%)	37(48.7%)	0.070
**Imaging**				
**Poor collaterals, n (%)**	53(30.6%)	15(15.5%)	38(50.0%)	<0.001^*^
**Cortical GCA, median (IQR)**	2(0-6)	2 (0-4)	4 (1.75-7)	0.009^*^
**Frontal atrophy**	0(0-2)	0(0-1.75)	0(0-2)	0.150
**Parieto-occipital atrophy**	1(0-2)	0(0-2)	2(0-2)	0.067
**Temporal atrophy**	2(0-2)	1(0-2)	2(0-3)	0.002^*^
**HT, n (%)**	71(41.0%)	29(29.9%)	42 (55.3%)	0.001^*^

*P<0.05. mTICI, modified Thrombolysis in Cerebral Infarction (mTICI) scale; SBP, systolic blood pressure; DBP, diastolic blood pressure; NIHSS, National Institute of Health Stroke Scale; ASPECTS, Alberta Stroke Program Early Computed Tomography Score; ICA, internal carotid artery; MCA, middle cerebral artery; FBG, fast blood glucose; LAA, large artery atherosclerosis; CE, cardioembolism; OTP, time interval from symptoms onset to puncture; OTR, time interval from symptoms onset to recanalization; GCA, global cortical atrophy; HT, hemorrhagic transformation.

**Figure 1.**
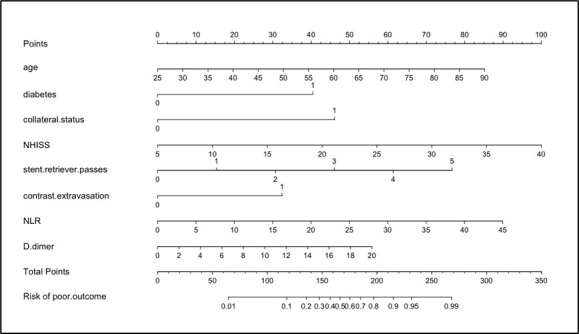
the comprehensive model exhibited by nomogram.

## RESULTS

Totally, 490 patients with AIS, secondary to anterior circulation vessels, and treated with EVT during January 2019 to June 2021 were screened, and 173 of them that achieved mTICI score of 3 after EVT were eligible for this study (Supplementary Fig. 1). Among the included patients, 106 of them (61.3%) were male and 67 of them (28.7%) were female. The mean age of was 65.3 ± 12.8 years, median admission NHISS was 15 (IQR 12-18), and median admission ASPECTS was 9 (IQR 7-10). Seventy-six patients (43.9%) experienced poor clinical outcome with mRS score of 3-6 at 3 months.

### Univariate analysis

Clinical and demographic characteristics are summarized in Table 1. Statistical differences (P<0.05) between patients with or without futile recanalization were documented in clinical variates including age, sex, diabetes, previous stroke, admission NHISS, and fast blood glucose.

Patients with futile recanalization had a higher rate of the stent retriever procedures (54 [71.1%] vs 52 [53.6%], *p*=0.019) and more passes of stent retriever (1 [IQR, 0-2] vs 1 [IQR, 0-1], *p*=0.006). Among patients with effective recanalization, 15 (15.5%) had poor collateral circulation, whereas 38 (50%) of patients with futile recanalization had poor collateral circulation *(p*<0.001). Patients with futile recanalization had higher cortical GCA scores (4, IQR 1.75-7) as compared with patients with effective recanalization (2, IQR 0-4, *p*=0.009). Hemorrhagic transformation was more commonly seen in patients with futile recanalization (42 [55.3%] vs 29 [29.9%], p=0.001).

Comparisons of potential no-reflow biomarkers between the two groups of patients are summarized in Table 2. Serum biomarkers of inflammation containing NLR, PLR, and SIRI were compared between two groups. Patients with futile recanalization had a higher level of NLR (6.02 [IQR 3.61-10.30] vs. 4.48 [IQR 2.75-6.63]), *p*=0.008 and a higher level of PLR (164.1 [IQR 122.3-253.2] vs. 149.2 [IQR 107.9-214.4], *p*=0.046) as compared with those with effective recanalization. Patients with futile recanalization also had higher levels of coagulation and fibrinolysis markers, including higher levels of D-dimer (1.91 [IQR 0.81-3.71] vs. 0.93 [IQR 0.35-2.29], *p*=0.001) and fibrinogen (3.48 [IQR 2.92-4.16] vs. 2.94 [IQR 2.54-3.59], *p*=0.002). 49 (64.5%) patients with futile recanalization experienced contrast extravasation while only 29 (29.9%) patients with effective recanalization suffered contrast extravasation (*p*<0.001), indicating that patients with futile recanalization were more likely to suffer from BBB disruption. No statistical differences were detected in platelet profile.

**Table 2:** Comparisons of no-reflow biomarkers between patients with effective or futile recanalization.

Factors	Total number (n=173)	Effective recanalization (mRS 0-2) (n=97)	Futile recanalization (mRS 3-6) (n=76)	P value
**Inflammation**				
WBC count, n*10^9^/L	8.72 (6.90-10.50)	8.27(6.80-10.27)	8.82(7.03-10.54)	0.373
Neutrophil count, n*10^9^/L	6.52 (4.90-8.47)	6.18 (4.76-8.26)	7.16 (4.92-9.23)	0.153
Lymphocyte count, n*10^9^/L	1.35 (0.99-1.85)	1.47 (1.11-1.90)	1.17 (0.83-1.62)	0.008^*^
Monocyte count, n*10^9^/L	0.39 (0.30-0.51)	0.41 (0.32-0.52)	0.27 (0.17-0.38)	0.192
**NLR, median (IQR)**	5.14 (3.05-8.08)	4.48(2.75-6.63)	6.02 (3.61-10.30)	0.008^*^
**PLR, median (IQR)**	156.9(111.5-221.8)	149.2(107.9-214.4)	164.1 (122.3-253.2)	0.046^*^
**SIRI, median (IQR)**	1.88(1.18-3.08)	1.80(1.10-2.35)	1.97 (1.21-3.67)	0.080
**Platelet profile**				
Platelet count, n*10^9^/L	213.51±63.51	216.39±60.18	209.83±67.76	0.502
**mean platelet volume, fl**	9.90(9.40-10.50)	9.90(9.40-10.50)	9.9(9.30-10.65)	0.647
**platelet distribution width, %**	10.60(9.70-12.15)	10.60(9.85-12.00)	10.50 (9.50-12.20)	0.870
**Coagulation and fibrinolysis**				
**D-dimer, ug/ml**	1.22(0.51-3.01)	0.93(0.35-2.29)	1.91 (0.81-3.71)	0.001^*^
**Fibrinogen, g/L**	3.24(2.66-3.86)	2.94(2.54-3.59)	3.48 (2.92-4.16)	0.002^*^
**BBB disruption**				
**Contrast extravasation, n (%)**	78(45.1%)	29(29.9%)	49 (64.5%)	<0.001^*^

*P<0.05. WBC, white blood cell; NLR, neutrophil-to-lymphocyte ratio; PLR, platelet-to-lymphocyte; SIRI, systemic inflammatory response index; BBB, blood-brain barrier.

### Multivariate analysis

All variables with a *p* value < 0.05 in the univariate analysis were included in the multivariate analysis. The basic model was constructed incorporating the clinical variables, and the comprehensive model was established by adding the no-reflow markers into the basic model. More detailed information is provided in Table 3. Results showed that age (OR, 1.061; 95% CI, 1.023-1.101; *p*=0.002), diabetes (OR, 6.220; 95% CI, 2.280-16.973; *p*<0.001), NHISS at admission (OR, 1.138; 95% CI, 1.042-1.242; *p*=0.004), poor collaterals (OR, 8.026; 95% CI, 2.920-22.052; *p*<0.001), passes of stent retriever (OR, 2.001; 95% CI, 1.248-3.206; *p*=0.004), contrast extravasation (OR, 4.321; 95% CI, 1.794-10.405; *p*=0.001), NLR (OR, 1.095; 95% CI, 1.009-1.188, *p*=0.030), and D-dimer (OR, 1.134; 95% CI, 1.017-1.266; *p*=0.024) were significantly associated with clinical outcomes in this patients population.

**Table 3:** Variables in basic and comprehensive models of futile recanalization.

Variable	Model 1 (without no-reflow biomarkers)			Model 2 (with no-reflow biomarkers)		
	OR	95%CI	P value	OR	95% CI	P value
**Age**	1.058	1.023-1.904	0.001	1.061	1.023-1.101	0.002
**Diabetes**	4.691	1.992-11.047	<0.001	6.220	2.280-16.973	<0.001
**NHISS at admission**	1.143	1.047-1.249	0.003	1.138	1.042-1.242	0.004
**Poor collaterals**	8.197	3.293-20.405	<0.001	8.026	2.920-22.052	<0.001
**Passes of stent retriever**	1.977	1.261-3.098	0.003	2.001	1.248-3.206	0.004
**Contrast extravasation**	-			4.321	1.794-10.405	0.001
**NLR**	-			1.095	1.009-1.188	0.030
**D-dimer**	-			1.134	1.017-1.188	0.024

NIHSS, National Institute of Health Stroke Scale; NLR, neutrophil-to-lymphocyte ratio.

**Figure 2.**
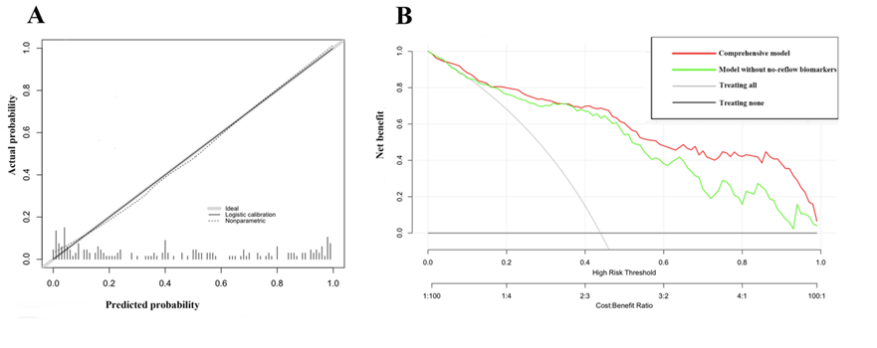
calibration plot and decision curve analysis. A, calibration plot; B, decision curve plot.

### Construction of the comprehensive model

A nomogram for predicting futile recanalization with poor functional outcomes at 90 days was constructed. As shown in Figure 1, the assigned points for each predictor were summed into total points. Higher total points were associated with an increased risk of poor outcome at 90 days for AIS patients achieving full reperfusion.

The calibration of the comprehensive model was confirmed by calibration plot in figure 2A which revealed good predictive accuracy. C-index that reflected discrimination of the nomogram was 0.901 (95%CI, 0.855-0.947), which demonstrating a good predictive power for this model. The model was internally validated by bootstraps of 1000 resamples with a C-index of 0.911 (95% CI, 0.863-0.954).

**Table 4:** Performance of models with potential markers of no-reflow to predict futile recanalization.

	AUC			IDI			NRI
**Model**	AUC (95%CI)	P value	Estimate% (95%CI)	P value	Estimate% (95%CI)	P value	
**Model 1**	0.868 (0.814-0.923)	Reference	Reference		Reference		
**Model 1 + NLR**	0.878 (0.824-0.932)	0.166	2.67 (0.41-4.93)	0.021	11.45 (1.23-21.67)	0.028	
**Model 1 + D-D**	0.876 (0.822-0.930)	0.306	2.71 (0.26-5.16)	0.030	4.69 (-4.55-13.93)	0.319	
**Model 1 + CE**	0.887 (0.838-0.936)	0.127	4.14 (0.85-7.43)	0.014	21.58 (7.45-35.72)	0.002	
**Model 2**	0.901 (0.855-0.947)	0.029	8.86 (4.34-13.39)	<0.001	18.38(3.53-33.23)	0.015	

* Patients were classified into 3 risk categories: 0% to 20%, 20% to 60%, and 60% to 1; Model-basic was constructed by clinical variates (age, NHISS, diabetes, poor collateral status, passes of stent retriever). NLR, neutrophil-to-lymphocyte ratio; CE, contrast extravasation. Model 1 was the model without no-reflow biomarkers and model 2 was the comprehensive model with no-reflow biomarkers.

### Additional predictive values of no-reflow potential markers

To confirm the additional predictive value of serum no-reflow biomarkers, AUC, IDI and categorized NRI were also calculated (Table 4). Compared to the model without no-reflow biomarkers, improved predictive performance was demonstrated when adding: NLR (AUC, 0.878 [95% CI, 0.824-0.932], *p*=0.166; IDI, 2.67% [95% CI, 0.41%-4.93%], *p*=0.021; NRI, 11.45% [95% CI, 1.23%-21.67%], *p*=0.028), D-dimer (AUC, 0.876 [95% CI, 0.822-0.930], *p*=0.306; IDI, 2.71% [95% CI, 0.26%-5.16%], *p*=0.030; NRI, 4.69% [95%CI, -4.55-13.93], *p*=0.319), contrast extravasation (AUC, 0.887 [95% CI, 0.838-0.936], *p*=0.127; IDI, 4.14% [95% CI, 0.85%-7.43%], *p*=0.014; NRI, 21.58% [95% CI, 7.45%-35.72%], *p*=0.002) and their incorporation (AUC, 0.901 [95% CI, 0.855-0.947], p=0.029; IDI, 8.86% [95% CI, 4.34%-13.39%], *p*<0.001; NRI, 18.38% [95% CI, 3.53%-33.23%], *p*=0.015) to the model.

Decision curve analysis was also performed in the comprehensive model and the model without these potential markers, respectively. As shown in figure 2B, when the threshold probabilities ranged from 4.4% to 100% in the comprehensive model, the nomogram showed a positive net benefit. But in the model without NLR, D-dimer and CE, the positive net benefit couldn’t show until the threshold probability was 10.7% and the net benefit was always lower compared with the comprehensive model.

## DISCUSSION

In this study, we developed a precise nomogram model to predict the occurrence probability of futile recanalization of AIS patients who achieving complete reperfusion, which may provide support to develop clinical strategies. The model contained clinical factors (age, diabetes, admission NHISS, passes of stent retriever), imaging biomarker (collateral status) and potential no-reflow biomarkers (NLR, D-dimer and contrast extravasation). And potential no-reflow biomarkers can greatly improve the predictive accuracy of the prediction model (AUC, 0.901 [95% CI, 0.855-0.947], p=0.029; IDI, 8.86% [95% CI, 4.34%-13.39%], p<0.001; NRI, 18.38% [95% CI, 3.53%-33.23%], p=0.015).

In this study, we have sought to build upon the existing body of knowledge regarding the prediction of futile recanalization following EVT in patients with AIS. Our findings are similar to those in recent years and have been expanded further [10]. While prior research has categorized mTICI 2b-3 as a marker of successful reperfusion, it is crucial to note that there are significant differences in clinical outcomes between mTICI 2b and mTICI 3, with the latter typically associated with more favorable prognoses [10]. Specifically, studies have shown that patients who achieve complete reperfusion (mTICI 3) through mechanical thrombectomy devices tend to have a better outcome than those who only reach a mTICI 2b level of reperfusion [27]. Acknowledging the critical role of achieving full recanalization, our research is concentrated on AIS patients who have reached an mTICI 3 score post-EVT. Recently proposed that additional research is required to more precisely identify patients at high risk of clinically futile reperfusion after EVT and to enhance personalized periprocedural management strategies to improve the chance of achieving favorable clinical outcomes [28].Our study specifically examines AIS patients who have achieved full recanalization following EVT treatment, with a perfusion degree of mTICI3. By focusing on this group, we aim to contribute to the body of research addressing the gap identified in the literature regarding the prediction and improvement of patient outcomes post-EVT.

Our study's multivariate logistic regression analysis identified several independent risk factors for futile recanalization after EVT, the patient's age, diabetes, NIHSS score at admission, poor collateral circulation, passes of stent retriever, NLR, D-Dimer and contrast extravasation. We constructed a nomograph model through these factors in the hope of accurately predicting the risk of futile recanalization. Previous studies have identified potential predictors of futile recanalization in AIS patients who achieved successful reperfusion (mTICI 2b-3), such as older age, vascular risk factors like smoking, arterial hypertension, dyslipidemia, and diabetes, higher NIHSS scores at admission, extended time to recanalization, and poor collateral circulation [10, 29].This is partly consistent with our nomogram model, age, diabetes, NIHSS score at admission and collateral circulation are our significant predictors. Our nomogram model corroborates these findings, with age, diabetes, NIHSS score at admission, and collateral circulation standing out as significant predictors in our analysis.

The pathophysiological mechanism underlying futile recanalization are multifaceted and include no-reflow phenomenon, microcirculatory disturbance, reperfusion injury, cerebral oedema formation. Notably, the no-reflow phenomenon is a critical factor that prevents the rescue of the ischemic penumbra and leads to the expansion of the core infarct area. By incorporating no-reflow biomarkers (NLR, D-Dimer and contrast extravasation) into our predictive model, we significantly improved the model's precision in predicting outcomes. This underscores the significant impact of the microvascular no-reflow phenomenon on functional recovery post-stroke.

Our research confirms the association between NLR and 3-month outcomes and mortality in AIS patients after EVT, supporting previous studies and emphasizing its prognostic value [30]. NLR represents the ability to regulate the immune system after stroke and can predict susceptibility to complications after stroke [31]. For example, a higher NLR can identify patients at an elevated risk of post-stroke infection, hemorrhagic transformation and neurological deterioration within the first 24 hours, enabling adjustment of patient nursing care strategies [32]. Consistent with Brooks et al, our nomogram highlights the importance of NLR in predicting the chances of futile recanalization [30].

The role of preoperative D-dimer levels identified by Baek et al. was confirmed in our study, with elevated levels predicting a predisposition to poor outcomes after EVT [33]. High D-dimer level reflects the existence of hard or fibrin-rich thrombus, which usually requires more passes of the thrombectomy devices and a longer time from groin puncture to recanalization [34, 35]. The results of this study are in line with them, further guiding neuro-interventionists to adjust their surgical strategies if they find that baseline D-dimer is elevated in AIS patients.

Operational factors, such as the number of stent retriever passes and contrast extravasation, have been implicated in futile recanalization. Our findings align with those of Baek et al., suggesting that exceeding a threshold number of retriever passes may not yield additional benefits and could instead extend the time to recanalization [36]. This underscores the need for a refined endovascular strategy that can indicate when to pivot from stent retrievers to alternative modalities for refractory large artery occlusions. Contrast extravasation, a marker of BBB disruption, has emerged as a strong prognostic indicator [37]. Our study adds to the understanding of its implications, highlighting the need for procedural strategies that mitigate BBB damage [38, 39], such as reducing recanalization time, careful delivery of EVT devices, and stringent post-EVT blood pressure control.

Diabetes, associated with microvascular dysfunction, is linked to BBB disruption and increased edema [40]. Our research indicates that glucose stabilization may be crucial in reducing these effects, potentially enhancing patient outcomes. Furthermore, the condition of collateral circulation significantly influences hemorrhagic transformation and prognosis following EVT [41]. Our findings highlight the significance of collateral circulation in determining infarct size and the efficacy of reperfusion treatments, underscoring the necessity for thorough preoperative assessments to refine therapeutic strategies. The nomogram serves as a sophisticated predictive model, adept at distilling complex risk factors into an intuitive scoring system for clinicians. By integrating pivotal predictors such as the NLR, preprocedural D-dimer levels, the number of stent retriever passes, and the presence of contrast extravasation, the nomogram offers a nuanced and scientifically grounded framework for estimating the likelihood of unsuccessful recanalization following EVT. Moreover, the parameters incorporated into the nomogram are readily accessible in the clinical setting, providing a wealth of valuable prognostic insights with ease and efficiency [42]. This feature enhances the practical utility of the nomogram, enabling its integration into routine clinical workflows. Consequently, the nomogram stands out as a user-friendly, precise, and trustworthy tool that can be employed to predict the occurrence of futile recanalization in patients with anterior circulation stroke who achieve complete recanalization post-EVT.

Our study introduces a predictive model for futile recanalization following EVT, but it is not without limitations. 1) The biomarkers introduced in this study are based on assumptions and require validation in human subjects. 2) The intricacies of the no-reflow phenomenon mean that we have not evaluated all pathophysiological pathways and their indicators. Future investigations should aim to validate these potential markers using sophisticated perfusion imaging methods in human subjects. 3) The study relies on internal validation of the predictive model. We emphasize that future studies should aim to externally validate the nomogram in different clinical settings to ensure its broader applicability. 4) Our study was conducted at a single high-volume stroke center; future research should focus on conducting multi-center studies to improve the generalizability of the findings. 5) This study is limited to the anterior circulation strokes, and the posterior circulation strokes should be included in the future to understand the EVT results more comprehensively.

## Conclusions

In conclusion, our research has effectively created a nomogram that combines clinical, laboratory, and imaging data to forecast the probability of futile recanalization in patients with anterior circulation AIS who attain complete recanalization post-EVT. Future endeavors will concentrate on validating this nomogram in various centers and expanding the patient cohort. The study only focuses on anterior strokes, but including posterior strokes could enhance understanding of EVT outcomes. Additional studies will also seek to identify independent risk factors for futile recanalization to enhance clinical decision-making and patient outcomes.

## Supplementary Materials

The Supplementary data can be found online at: www.aginganddisease.org/EN/10.14336/AD.2024.0127.
