# Supplementary material for: A Comprehensive Prediction Model for Futile Recanalization in AIS Patients Post-Endovascular Therapy: Integrating Clinical, Imaging, and No-Reflow Biomarkers
Source: Aging Dis. 2024 Apr 25;15(6):2852–62. doi: 10.14336/AD.2024.0127 (PMC11567269; doi:10.14336/AD.2024.0127)
Supplement: Supplementary file 1 — The Supplementary data can be found online at: www.aginganddisease.org/EN/10.14336/AD.2024.0127. [file AD-15-6-2852-s.pdf]

SUPPLEMENTARY DATA

A Comprehensive Prediction Model for Futile Recanalization in AIS Patients Post-Endovascular Therapy: Integrating Clinical, Imaging, and No-Reflow Biomarkers

Shuangfeng Huang, Jiali Xu, Haijuan Kang, Wenting Guo, Changhong Ren, Alexandra Wehbe, Haiqing Song, Qingfeng Ma, Wenbo Zhao, Yuchuan Ding, Xunming Ji, Sijie Li

© 2024. Huang S et al. Published online at http://www.aginganddisease.org/EN/10.14336/AD.2024.0127

SUPPLEMENTARY DATA

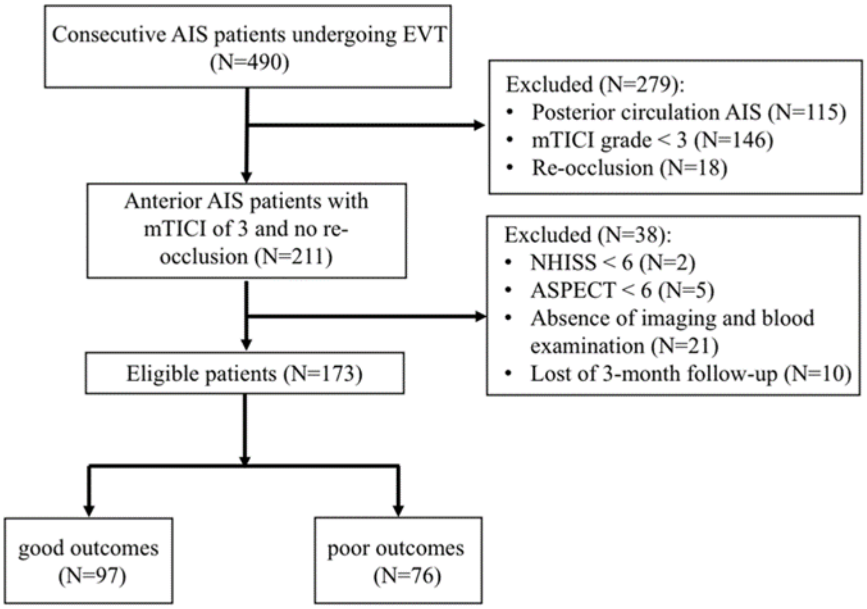

Supplementary Figure 1. Flow chart.

Supplementary Table 1. Referential intervals of blood biomarkers.

Blood biomarker	referential intervals
**Inflammation**	
WBC, n*10^9/L	4-10
Neutrophil count, n*10^9/L	1.8-6.4
Lymphocyte count, n*10^9/L	1-3.3
Monocyte count, n*10^9/L	0.2-0.7
**Platelet profile**	
Platelet count, n*10^9/L	100-300
mean platelet volume, fl	9.4-12.5
platelet distribution width, %	0-15
**Coagulation and fibrinolysis**	
D-dimer, ug/ml	0.01-0.5
Fibrinogen, g/L	2-4

WBC, white blood cell; NLR, neutrophil-to-lymphocyte ratio; PLR, platelet-to-lymphocyte; SIRI, systemic inflammatory response index; LDL, low density lipoprotein

© 2024. Huang S et al. Published online at http://www.aginganddisease.org/EN/10.14336/AD.2024.0127
